# Closed-Loop Insulin Delivery Systems: Past, Present, and Future Directions

**DOI:** 10.3389/fendo.2022.919942

**Published:** 2022-06-06

**Authors:** Sophie Templer

**Affiliations:** Department of Endocrinology, St Vincent’s Hospital, Sydney, NSW, Australia

**Keywords:** artificial pancreas, closed-loop systems, glycemic control, type 1 diabetes, medical devices

## Abstract

Closed-loop (artificial pancreas) systems for automated insulin delivery have been likened to the holy grail of diabetes management. The first iterations of glucose-responsive insulin delivery were pioneered in the 1960s and 1970s, with the development of systems that used venous glucose measurements to dictate intravenous infusions of insulin and dextrose in order to maintain normoglycemia. Only recently have these bulky, bedside technologies progressed to miniaturized, wearable devices. These modern closed-loop systems use interstitial glucose sensing, subcutaneous insulin pumps, and increasingly sophisticated algorithms. As the number of commercially available hybrid closed-loop systems has grown, so too has the evidence supporting their efficacy. Future challenges in closed-loop technology include the development of fully closed-loop systems that do not require user input for meal announcements or carbohydrate counting. Another evolving avenue in research is the addition of glucagon to mitigate the risk of hypoglycemia and allow more aggressive insulin dosing.

## Introduction

The mainstay of treatment for type 1 diabetes is intensive insulin therapy, either as multiple daily injections or continuous subcutaneous insulin infusion *via* pump. The goal of intensive insulin therapy is to mimic physiological insulin release by pancreatic beta cells in a basal-bolus fashion to achieve tight glycemic control and thereby reduce the risk of micro- and macrovascular complications of hyperglycemia (1). However, optimal glycemic control in many individuals with type 1 diabetes is limited by hypoglycemia and the high burden of self-management required with frequent monitoring of blood glucose and adjustment of insulin dosing (2). As a result, a majority of people with type 1 diabetes are unable to achieve the recommended therapeutic targets (3).

In the 100 years since the discovery of insulin, there have been significant technological advances in diabetes management. Insulin pumps first became clinically feasible in the 1970s, and have since become miniaturized and more reliable. Continuous glucose monitoring systems (CGMS) are now minimally invasive and more accurate. There is a growing demand for connection of these two types of devices with algorithms that can facilitate automated insulin delivery. These closed-loop systems – also referred to as the “artificial pancreas” – have been likened to the holy grail of diabetes management as they have the potential to improve glycemic outcomes and reduce disease burden (4).

## Early Closed-Loop Systems

The first closed-loop insulin delivery system was developed by Arnold Kadish in the early 1960s. Kadish’s invention, which he termed a “servomechanism for blood glucose control”, comprised an autoanalyzer for continuous blood glucose monitoring *via* an intravenous catheter and two intravenous syringe pumps containing insulin and either glucose or glucagon. Both pumps were shut off when the blood glucose level was within a defined target range; the insulin pump was activated when the glucose level rose above the upper threshold, and the glucose or glucagon pump was activated when it dropped below the lower threshold (5, 6). Kadish published the results of a successful trial of his system in a single diabetic volunteer in 1963 (5).

**Figure 1:**
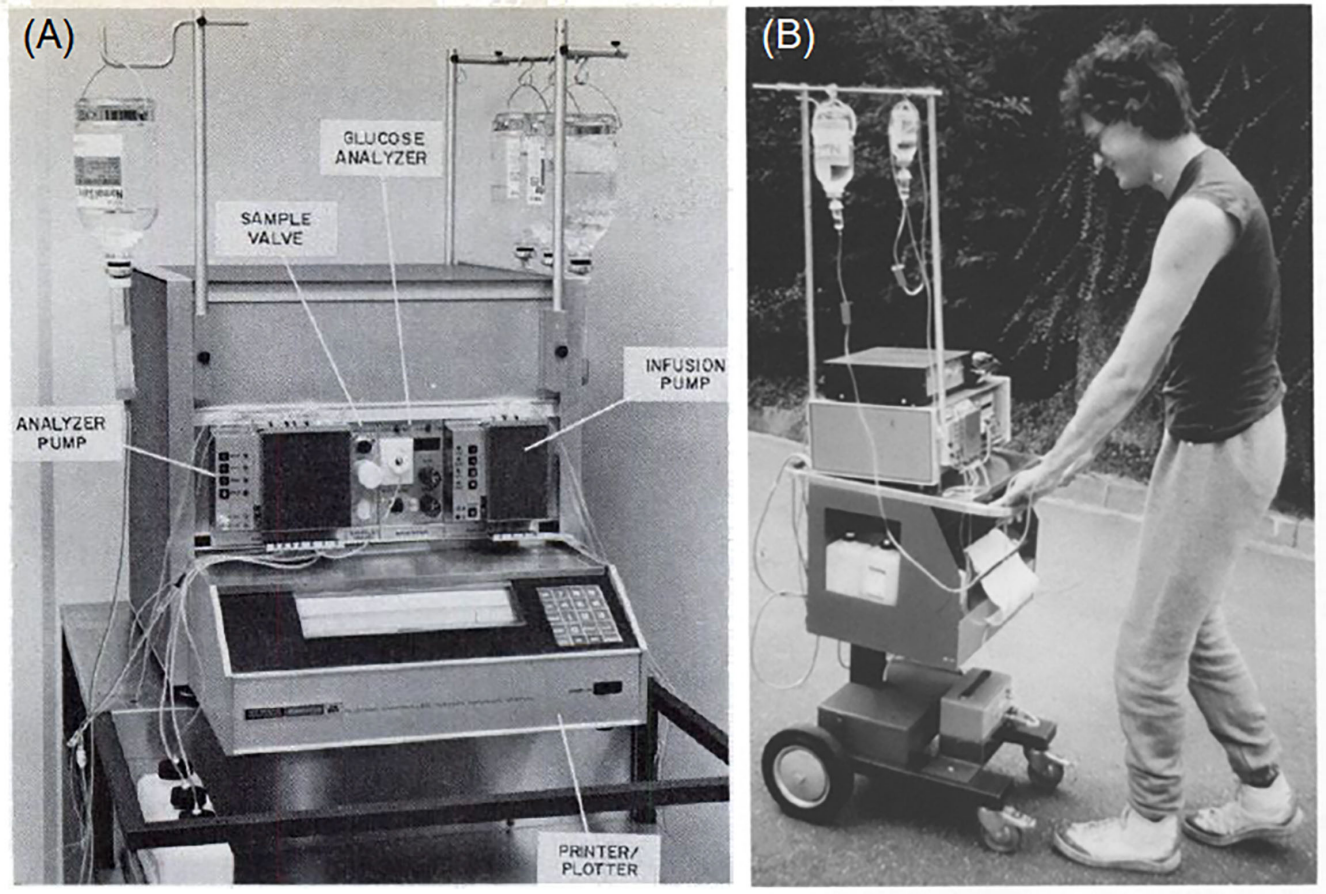
Early closed-loop technologies: **(A)** Components of the Biostator (reproduced from Fogt et al., 1978); **(B)** A mobile version of the Biostator (reproduced from Pfeiffer 1987) (10, 11).

The first systems to be described as an artificial pancreas were developed in the early 1970s. Albisser and colleagues (a Canadian group) and Pfeiffer and colleagues (a German group) separately designed essentially the same configuration of apparatuses and both published their findings in 1974 (7–9). Both systems utilized a computer programmed to respond to continuous venous glucose monitoring and control the intravenous delivery of insulin and/or dextrose. The apparatus originally developed by Pfeiffer et al. (**Figure 1**) was commercialized in 1977 as the Biostator (Miles Laboratories, Elkhart, Indiana, USA), which consisted of: a pump which controlled continual blood withdrawal; a glucose analyzer for continuous measurement of blood glucose concentration; a computer programmed to calculate the amount of insulin or dextrose to be infused based on blood glucose levels; an infusion pump for insulin and dextrose delivery; and a printer for minute-by-minute blood glucose recording (10, 12).

Because the Biostator was bulky, intricate, and required the patient to be connected to a blood withdrawal catheter in one arm and an infusion line in the other arm, its use was largely limited to research. It was also employed as an investigative tool to study an individual’s glycemic patterns over a 24–36 hour hospital admission, in order to help determine their ideal insulin dosage (13). The Biostator was used extensively in research throughout the 1980s and 1990s, with over 200 publications based upon its use (14).

The first wearable artificial pancreas system was developed by a Japanese group led by Motoaki Shichiri in the early 1980s (15, 16). The whole system, consisting of a sensor, a microcomputer and two roller pumps, weighed 400 grams and measured 15 x 12 x 6 cm, and was able to be stored in the pocket of the user’s jacket. While the Biostator was an intravenous-intravenous system, using venous glucose sensing and intravenous insulin delivery, Shichiri’s technology used a subcutaneous glucose sensor paired with intravenous pumps for insulin and glucagon infusions (16).

## Current Closed-Loop Technologies

**Figure 2:**
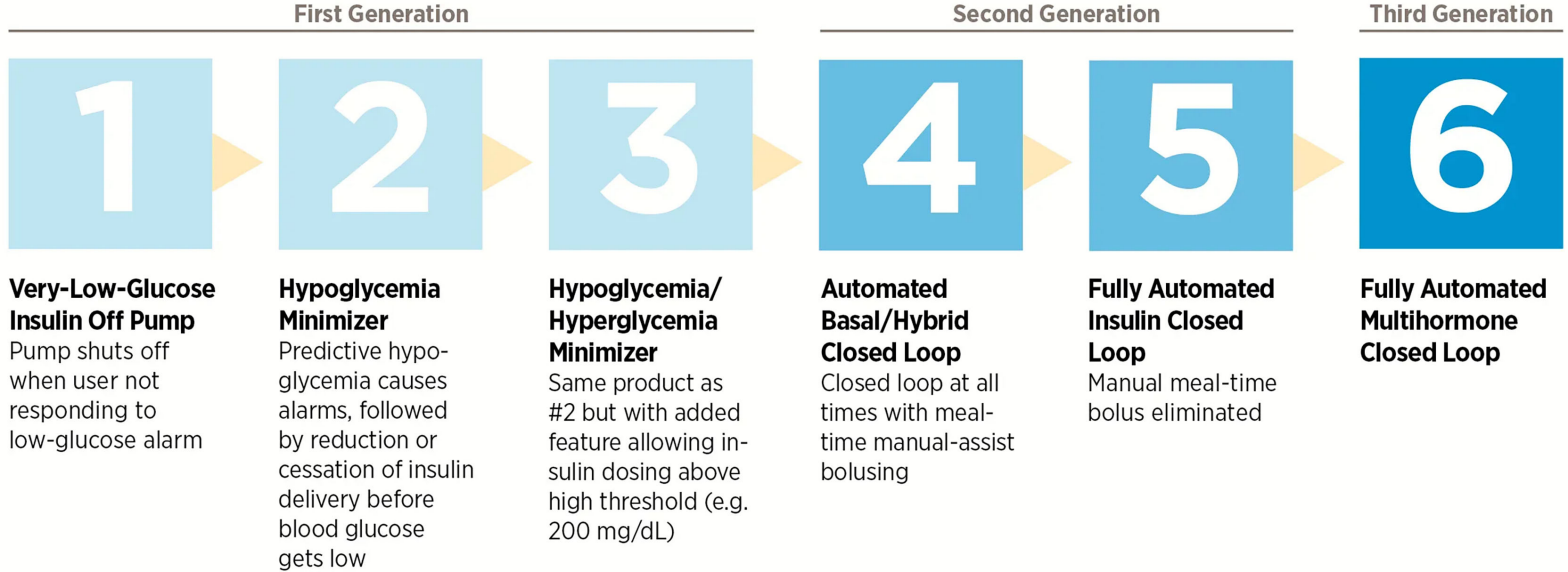
The six categories of closed-loop systems as defined by the JDRF.

Progress towards a fully closed-loop system have been accelerating since the mid-2000s, with the development and commercialization of numerous glucose-sensing and insulin delivery systems of increasing sophistication. With the technological progress made regarding insulin pumps and interstitial glucose-sensing devices, attention turned to the development of a subcutaneous-subcutaneous closed-loop system (17). The Juvenile Diabetes Research Foundation (JDRF) established the Artificial Pancreas Project in 2005 with the aim of promoting the research, regulatory approval, and eventual adoption of closed-loop technologies (4). The JDRF defined six categories of artificial pancreas systems based on the level of automation involved (**Figure 2**); at the time, all were in varying stages of development but none were commercially available.

### Low-Glucose Suspend Systems

Low-glucose suspend (LGS) systems are the simplest form of a closed-loop system. They consist of an integrated glucose sensor and insulin pump with the ability to automatically suspend insulin infusion when glucose levels fall below a certain threshold without requiring any confirmation from the user. In 2009, Medtronic commercialized the first LGS system with the MiniMed Paradigm Veo (Medtronic, Northridge, California, USA), which suspends insulin delivery and alerts the user when a pre-programmed glucose threshold is reached (18). The primary benefit of LGS over sensor-augmented pump therapy is reduced nocturnal hypoglycemia, without an increase in HbA1c (19, 20).

LGS technology was further refined in the form of predictive low-glucose suspend (PLGS) systems, which contain algorithms that predict future hypoglycemia (for example, within the next 30 minutes) and pre-emptively suspend insulin delivery before hypoglycemia occurs. This technology became commercially available in 2015 with the MiniMed 640G (Medtronic), and can also be found in the t:slim X2 with Basal-IQ (Tandem, San Diego, California, USA). Like LGS, use of PLGS is associated with a significantly reduced risk of nocturnal hypoglycemia as well as overall time spent in hypoglycemia, without an increase in hyperglycemia (21, 22).

### Hybrid Closed-Loop Systems

Hybrid closed-loop systems aim to minimize hypoglycemia and hyperglycemia and maintain glucose levels within a target range through the use of a computerized algorithm to adjust the basal rate of insulin and administer corrective bolus doses. They are called “hybrid” systems as, unlike fully closed-loop systems, the user is still required to manually program insulin boluses with meals. Development of the first hybrid closed-loop systems began in parallel with LGS technology. The Advanced Insulin Infusion Using a Control Loop (ADICOL) project was launched in 2000, with the collaboration of several European centers to develop one of the first hybrid closed-loop systems (23). A pivotal trial by Weinzimer et al. in 2008 was the first to show that a hybrid closed-loop system significantly improved overnight time spent in the normoglycemic range compared to conventional open-loop insulin delivery (24). Further trials in adult and pediatric populations have demonstrated increased time in target and reduced hypoglycemia, mean glucose levels, and HbA1c in hybrid closed-loop systems (25–28).

**Figure 3:**
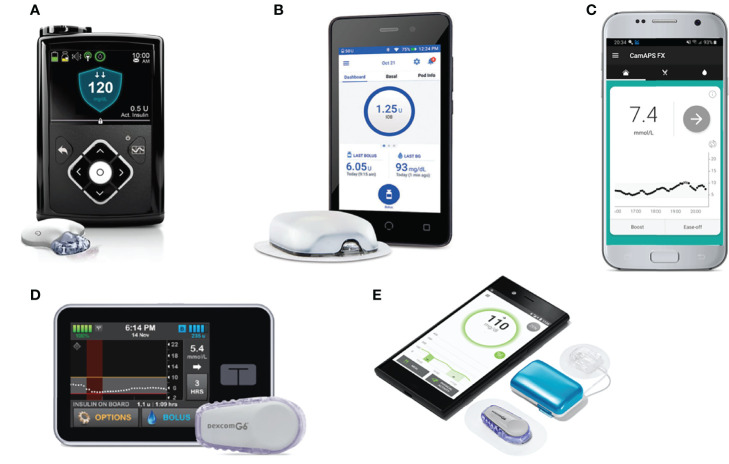
Commercially available and in-development hybrid closed-loop systems. **(A)** MiniMed 670G with Guardian Link 3 sensor/transmitter. **(B)** Omnipod Horizon with patch-pump. **(C)** CamAPS FX algorithm hosted on Android. **(D)** Tandem t:slim X2 pump paired with Dexcom G6 sensor. **(E)** Diabeloop DLBG1 algorithm with Kaleido patch-pump and Dexcom G6 sensor.

The MiniMed 670G (Medtronic), the first commercially available hybrid closed-loop system, was released in 2016. Other systems that have received regulatory approval (**Figure 3**) include the MiniMed 780G (Medtronic), t:slim X2 with Control-IQ (Tandem), and CamAPS FX (CamDiab, Cambridge, UK) (29). These systems use three main types of algorithms: model predictive control (MPC), proportional-integral-derivative (PID), and fuzzy logic. MPC algorithms use a mathematical model of the user’s glucoregulatory system to predict glucose excursions and adjust insulin delivery to treat-to-target, taking into account estimated insulin sensitivity. PID algorithms adjust insulin delivery according to three elements: the difference between measured and target glucose levels (the proportional component), the area under the curve between measured and target glucose (the integral component), and the rate of change in measured glucose levels over time (the derivative component). Algorithms based on fuzzy logic are less common, and modulate insulin delivery according to a set of rules designed to imitate the knowledge and reasoning of experienced diabetes clinicians (30).

The pivotal trial establishing the efficacy of the MiniMed 670G system was published by Garg et al. in 2017. The prospective analysis of 124 adults and adolescents using the system at home over three months demonstrated a significantly increased time in range compared to baseline (28). A later trial by Forlenza et al. in children aged 7–13 similarly found that in-home use of the MiniMed 670G resulted in increased time in range and reduced HbA1c compared to baseline (31). A prospective study by Lal et al. of real-world use of the MiniMed 670G over 12 months found significant correlation between time spent in Auto Mode (in which the hybrid closed-loop algorithm is activated) and HbA1c, but this was countered by a high discontinuation rate, with 33% of users having discontinued Auto Mode use by 12 months. The most frequent reasons reported for discontinuation included sensor issues, problems obtaining supplies, and fear of hypoglycemia (32). A recent retrospective analysis of data uploaded over a 15-month period by 14,899 European users of the MiniMed 670G found that users spent a mean 81.4% of the time in Auto Mode and could expect to spend 72% of the time in range with Auto Mode enabled, an increase of 10% compared with pre-Auto Mode initiation (33).

### Do-It-Yourself Closed-Loop Systems

The “do-it-yourself” (DIY) closed-loop movement began to gain momentum in 2013 when a group of people with type 1 diabetes and their families began collaborating online to create open-source closed-loop software. Many shared their knowledge and experiences under the hashtag #WeAreNotWaiting in reference to their frustration with the slow progress of medical device development and delays in regulatory approval of closed-loop systems (34, 35). These DIY systems connect commercially available insulin pumps and CGMS to an open-source algorithm, held either in a smartphone application or custom hardware, that analyses glucose data from the sensor and remotely adjusts insulin delivery by the pump. The first DIY closed-loop system contained a radio stick to communicate between the insulin pump and a minicomputer holding the algorithm, but the emergence of Bluetooth-enabled pumps means that an increasing number of these systems use smartphones or other mobile devices to host the algorithm and communicate directly with the pump. While most DIY systems operate similarly to conventional hybrid closed-loop systems, where users manually administer boluses with meals, some users choose to enable features that allow them to skip meal announcements and boluses (34).

Reliable figures of usage are difficult to track but recent estimates suggest that there are over 2000 worldwide users of DIY closed-loop systems including OpenAPS, AndroidAPS and Loop (36). The most attractive features of these systems for users include their low-cost availability and increased customizability compared to commercial hybrid closed-loop systems. Although few clinical trials have been conducted on DIY closed-loop systems, analyses of self-reported data from users have shown benefits in HbA1c, time in range, glucose variability, and fewer episodes of hypoglycemia. Reported quantitative outcomes include reduced mental burden of diabetes management and reduced reliance on carbohydrate counting (37). Objective comparison of data between patients is limited by the highly individualized use of DIY systems between users and the fact that they use open-source software, meaning each user can customize the algorithms. *In silico* studies may overcome this challenge, and have been used by some groups to establish the safety and efficacy of these systems, as well as providing comparison to commercialized technologies (38, 39); indeed, research on many commercially available closed-loop systems began with *in silico* trials (40).

Currently, practitioners are placed in a challenging position when caring for patients who are actively using or interested in using DIY systems. On the one hand, many patients report improvements in glycemic control and quality of life; on the other, these technologies lack formal safety studies and approval from regulatory bodies, and often involve off-label use of approved CGMS and insulin pumps (41).

## Future Directions in Closed-Loop Technology

**Figure 4:**
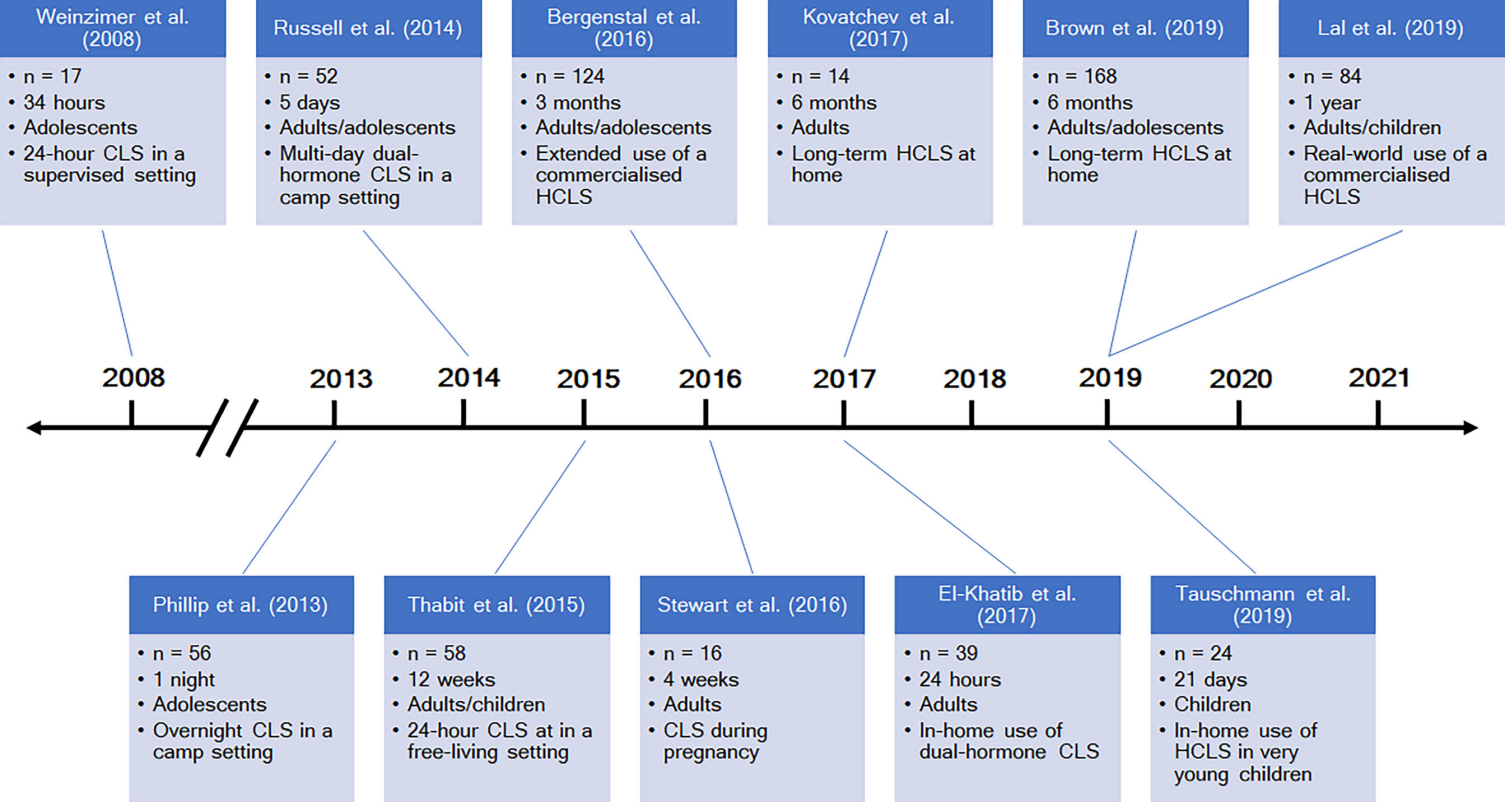
A timeline of selected studies of closed-loop systems. CLS = closed-loop system; HCLS = hybrid closed-loop system. References: Weinzimer et al., (24), Phillip et al., (42), Russell et al., (43), Thabit et al., (26), Bergenstal et al., (27), Stewart et al., (44), Kovatchev et al., (45), El-Khatib et al., (46), Brown et al., (47), Tauschmann et al., (48), Lal et al., (32).

The past five to ten years have seen an explosion in research and published literature about closed-loop systems (selected notable publications are highlighted in **Figure 4**). Multiple further hybrid systems are expected to be commercialized in the near future, in addition to those already available. The DBLG1 (Diabeloop, Grenoble, France) has received the CE mark in Europe for use in adults with type 1 diabetes, while the Omnipod Horizon (Insulet, Billerica, Massachusetts, USA) and insulin-only iLet (Beta Bionics, Boston, Massachusetts, USA) are currently undergoing clinical trials (49). On the DIY front, Tidepool, the non-profit software organization responsible for Loop, has submitted an application to the United States Food and Drug Administration (FDA) with the aim of releasing Loop as an FDA-regulated mobile application, supported by funding from the JDRF (50). Future directions in closed-loop research are principally aimed at the advanced generations of closed-loop systems as outlined by the JDRF: fully automated and multi-hormone systems.

### Fully Closed-Loop Systems

Fully closed-loop systems, unlike hybrid systems, are designed to automate all insulin delivery without requiring user input for mealtime boluses. The main challenge in fully closed-loop systems therefore is postprandial hyperglycemia, as there is no manually provided information about the timing and carbohydrate content of meals. These postprandial glucose excursions are often followed by hypoglycemia secondary to the delayed action of current rapid-acting insulins. Fully closed-loop systems can use the same types of algorithms as hybrid systems – MPC, PID, or fuzzy logic – although all fully closed-loop systems included in a 2017 meta-analysis used MPC-based algorithms (51). Investigators have made use of different algorithms to recognize unannounced meals and estimate carbohydrate intake based on either the rate of change in glucose levels or the required insulin boluses (52). Another proposed solution to mitigate postprandial glucose excursions is the integration of GoCARB, a smartphone application that estimates carbohydrate content based on user-submitted images of meals in real time, into an MPC algorithm (53).

An early trial by Kovatchev et al. in 2010 found that use of a fully closed-loop system in adults with type 1 diabetes improved hypoglycemia and time in target range compared to sensor-augmented pump therapy (54). Phillip et al. similarly showed a reduced incidence of hypoglycemia in a pediatric population using a fully closed-loop system (42). However, postprandial glucose excursions remain the largest limitation of fully closed-loop systems in direct comparisons with hybrid systems. In the pioneering study by Weinzimer et al., manual pre-meal insulin boluses reduced peak postprandial glucose excursions and mean daytime glucose compared to a fully closed-loop system (23). Forlenza et al. similarly found an improvement in postprandial hyperglycemia and mean glucose levels with manual mealtime boluses in a closed-loop system (55). Still, fully closed-loop systems may be suited for users who frequently miss or miscalculate mealtime boluses (56, 57).

Another challenge for fully closed-loop systems is glycemic control during and after exercise. An ideal algorithm would account not only for changes in glucose levels associated with exercise, but also the duration, intensity, and type of physical activity. Biometric data such as heart rate, skin temperature, accelerometry, and energy expenditure have been used in trials of a fully closed-loop system to recognize different types and intensities of exercise without any manual inputs (58). A feasibility study by Breton et al. showed that a heart rate monitor can be integrated into a wireless closed-loop system, although their exercise algorithm did not result in a significant reduction in hypoglycemic events (59).

Currently, the only commercially available fully closed-loop system is the STG-55 (Nikkiso, Tokyo, Japan) and its predecessor, the STG-22. As opposed to the more widely available wearable hybrid technologies, these are bedside devices that use intravenous-intravenous access for glucose sensing and insulin delivery. The STG-55 is only available in Japan, where its approval is limited to the perioperative setting for a maximum of a three-day period (60).

### Dual-Hormone Closed-Loop Systems

Two of the earliest closed-loop systems – those developed by Kadish and Shichiri – utilized a dual-hormone approach with a combination of insulin to counter hyperglycemia and glucagon to counter hypoglycemia. However, the use of glucagon in closed-loop systems fell out of practice in the Biostator era and first appeared in subcutaneous closed-loop systems in research in the mid-2000s (61). The primary rationale for dual-hormone systems, which are capable of administering boluses of glucagon in addition to continuous insulin infusion, is that prevention of hypoglycemia is more effective with administration of glucagon than with suspension of insulin delivery. This is due to the pharmacokinetics of subcutaneous insulin and glucagon: currently available rapid-acting insulins have a relatively slow onset (10–15 minutes), delayed time to maximum effect (40–60 minutes) and prolonged duration of action (up to 4–6 hours), while glucagon has an onset of 5 minutes (62).

There are two main approaches to insulin-glucagon systems: the first utilizes small boluses to prevent hypoglycemia without a concomitant increase in insulin delivery, while the second uses intermittent glucagon doses to allow more aggressive insulin delivery to target lower glucose levels (62). Compared with conventional insulin pump therapy, dual-hormone closed-loop systems have been shown to reduce hypoglycemia, improve mean glucose levels, and increase time spent in the target glycemic range (43, 63). A 2017 meta-analysis comparing single-hormone and dual-hormone closed-loop systems showed that the dual-hormone approach resulted in increased time in target (51). The main barrier to the development and uptake of glucagon-containing closed-loop systems is the lack of stable liquid formulations of glucagon; some studies have used glucagon cartridges that require replacement as frequently as every 8 hours (64). Recently, Castellanos et al. have published preliminary results from a trial of the dual-chamber iLet (Beta Bionics), which contains insulin and dasiglucagon, a chemically stable synthetic glucagon analogue (65).

Another dual-hormone approach combines insulin with pramlintide, a synthetic analogue of amylin, which is co-secreted with insulin by healthy pancreatic beta cells and slows gastric emptying, suppresses glucagon production, and prolongs satiety. One study showed in 2016 that the addition of fixed-dose premeal injections of pramlintide to a closed-loop system reduced postprandial hyperglycemia (66). Another trial demonstrated improved daytime glycemic control in a dual-hormone closed-loop system with basal-bolus delivery of pramlintide compared to an insulin-only closed-loop system (67). The practicality of insulin-pramlintide closed-loop systems is limited by the requirement for two separate infusion reservoirs, but this remains an area of ongoing research (68).

### Specific Populations

The safety and efficacy of several closed-loop systems have been established in large trials of adults and adolescents with type 1 diabetes in both controlled environments and real-life settings. However, there are many subpopulations who stand to benefit from closed-loop therapy. In the framework of personalized precision medicine, closed-loop control has the potential for success in individuals with unique physiological, pathological, and behavioral characteristics that influence glycemic control, such as pregnant women, very young children, critical care patients, dialysis patients, shift workers, and travelers. Most commercially available hybrid closed-loop systems are licensed for use in children, albeit with varying minimum ages for use (69). CamAPS FX is the only system currently licensed for use in pregnancy, although there are case reports of off-label use of the MiniMed 670G by pregnant women (70, 71). A significant barrier to closed-loop use during pregnancy is the need for a customizable algorithm that allows for adjustment of glycemic targets to the tighter range recommended in pregnancy.

A study of day-and-night hybrid closed-loop control during pregnancy by Stewart et al. found reduced hypoglycemia compared to sensor-augmented pump therapy, but no difference in the primary outcome of overall time spent in range (72). Bally et al. compared a similar hybrid system to conventional subcutaneous insulin therapy in hospitalized patients with type 2 diabetes, finding reduced hypoglycemia, reduced mean glucose, and increased time in range (73). A *post-hoc* analysis of this data focusing on patients undergoing hemodialysis similarly found an increased proportion of time in target and reduced hypoglycemia (74). A recent randomized trial of hybrid closed-loop therapy in children aged 1 to 7 demonstrated significant improvements in time in range, HbA1c, and mean glucose level compared to sensor-augmented pump therapy, without a significant difference in total daily insulin dose (75).

## Conclusion

Since the era of the first closed-loop systems in the 1960s and ‘70s, progress in diabetes management has been closely tied to advances in diabetes technology with the proliferation of devices for continuous insulin delivery and glucose monitoring. The past decade has seen rapid advances in the development and uptake of closed-loop systems, with the hybrid closed-loop system transitioning from research to commercial availability. Although the ultimate artificial pancreas – a fully closed-loop system – has not yet been realized in clinical practice, the success of closed-loop system development thus far, and the timeline in which it has been achieved, is promising.

The key open questions in closed-loop system development surround the capability of sensors, pumps, and algorithms to adapt to complex scenarios. Current technologies often struggle to handle glycemic dysregulation resulting from features of everyday life such as exercise, sleep disruption, and variable meal times and sizes. Will this require better sensors, without the built-in delay of interstitial glucose readings? Faster-acting insulins or alternative routes for insulin delivery, allowing for more rapid onset and offset? The addition of glucagon or other adjuncts to mitigate the risk of hypoglycemia? Or more advanced algorithms that can address not only person-to-person variability but also day-to-day variability in glucose regulation? The answers lie in the next generation of closed-loop therapy, which may well use a combination of these.

## Author Contributions

The author confirms being the sole contributor to this work and has approved it for publication.

## Conflict of Interest

The authors declares that the research was conducted in the absence of any commercial or financial relationships that could be construed as a potential conflict of interest.

## Publisher’s Note

All claims expressed in this article are solely those of the authors and do not necessarily represent those of their affiliated organizations, or those of the publisher, the editors and the reviewers. Any product that may be evaluated in this article, or claim that may be made by its manufacturer, is not guaranteed or endorsed by the publisher.

## References

[1] Diabetes Control and Complications Trial Research Group Nathan DM Genuth S Lachin J Cleary P Crofford O. The Effect of Intensive Treatment of Diabetes on the Development and Progression of Long-Term Complications in Insulin-Dependent Diabetes Mellitus. N Engl J Med (1993) 329:977–86. doi: 10.1056/NEJM199309303291401 8366922

[2] The DCCT Research Group. Epidemiology of Severe Hypoglycemia in the Diabetes Control and Complications Trial. Am J Med (1991) 90:450–59. doi: 10.1016/0002-9343(91)90605-W 2012085

[3] Foster NC Beck RW Miller KM Clements MA Rickels MR DiMeglio LA. State of Type 1 Diabetes Management and Outcomes From the T1D Exchange in 2016-2018. Diabetes Technol Ther (2019) 21:66–72. doi: 10.1089/dia.2018.0384 30657336 PMC7061293

[4] Hovorka R. Closed-Loop Insulin Delivery: From Bench to Clinical Practice. Nat Rev Endocrinol (2011) 7:385–95. doi: 10.1038/nrendo.2011.32 21343892

[5] Kadish AH. Automation Control of Blood Sugar a Servomechanism for Glucose Monitoring and Control. Trans Am Soc Artif Intern Organs (1963) 9:363–67. 13958209

[6] Kadish AH. A Servomechanism for Blood Sugar Control. BioMed Sci Instrum (1963) 1:171–76. 14174484

[7] Albisser AM Leibel BS Ewart TG Davidovac Z Botz CK Zingg W. An Artificial Endocrine Pancreas. Diabetes (1974) 23:389–96. doi: 10.2337/diab.23.5.389 4598089

[8] Albisser AM Leibel BS Ewart TG Davidovac Z Botz CK Zingg W. Clinical Control of Diabetes by the Artificial Pancreas. Diabetes (1974) 23:397–404. doi: 10.2337/diab.23.5.397 4598090

[9] Pfeiffer EF Thum C Clemens AH. The Artificial Beta Cell–a Continuous Control of Blood Sugar by External Regulation of Insulin Infusion (Glucose Controlled Insulin Infusion System). Horm Metab Res (1974) 6:339–42. doi: 10.1055/s-0028-1093841 4607598

[10] Fogt EJ Dodd LM Jenning EM Clemens AH. Development and Evaluation of a Glucose Analyzer for a Glucose Controlled Insulin Infusion System (Biostator). Clin Chem (1978) 24:1366–72. doi: 10.1093/clinchem/24.8.1366 679460

[11] Pfeiffer EF. On the Way to the Automated (Blood) Glucose Regulation in Diabetes: The Dark Past, the Grey Present and the Rosy Future. Diabetologia (1987) 30:51–65. doi: 10.1007/BF00274572 3552826

[12] Clemens AH Chang PH Myers RW. The Development of Biostator, a Glucose Controlled Insulin Infusion System (GCIIS). Horm Metab Res (1977) Suppl 7:23–33. 873440

[13] Young A Herf S. Biostator Glucose Controller: A Building Block of the Future. Diabetes Educ (1984) 10:11–2. doi: 10.1177/014572178401000203 6378557

[14] Lal RA Ekhlaspour L Hood K Buckingham B. Realizing a Closed-Loop (Artificial Pancreas) System for the Treatment of Type 1 Diabetes. Endocr Rev (2019) 40:1521–46. doi: 10.1210/er.2018-00174 PMC6821212 31276160

[15] Shichiri M Kawamori R Yamasaki Y Hakui N Abe H. Wearable Artificial Endocrine Pancreas With Needle-Type Glucose Sensor. Lancet (1982) 2:1129–31. doi: 10.1016/S0140-6736(82)92788-X 6128452

[16] Shichiri M Kawamori R Hakui N Yamasaki Y Abe H. Closed-Loop Glycemic Control With a Wearable Artificial Endocrine Pancreas. Variations in Daily Insulin Requirements to Glycemic Response. Diabetes (1984) 33:1200–02. doi: 10.2337/diab.33.12.1200 6389235

[17] Hanaire H. Continuous Glucose Monitoring and External Insulin Pump: Towards a Subcutaneous Closed Loop. Diabetes Metab (2006) 32:534–38. doi: 10.1016/S1262-3636(06)72808-7 17130814

[18] Agrawal P Welsh JB Kannard B Askari S Yang Q Kaufman FR. Usage and Effectiveness of the Low Glucose Suspend Feature of the Medtronic Paradigm Veo Insulin Pump. J Diabetes Sci Technol (2011) 5:1137–41. doi: 10.1177/193229681100500514 PMC3208869 22027306

[19] Bergenstal RM Klonoff DC Garg SK Bode BW Meredith M Slover RH. Threshold-Based Insulin-Pump Interruption for Reduction of Hypoglycemia. N Engl J Med (2013) 369:224–32. doi: 10.1056/NEJMoa1303576 23789889

[20] Ly TT Nicholas JA Retterath A Lim EM Davis EA Jones TW. Effect of Sensor-Augmented Insulin Pump Therapy and Automated Insulin Suspension vs Standard Insulin Pump Therapy on Hypoglycemia in Patients With Type 1 Diabetes: A Randomized Clinical Trial. JAMA (2013) 310:1240–47. doi: 10.1001/jama.2013.277818 24065010

[21] Forlenza GP Li Z Buckingham BA Pinsker JE Cengiz E Wadwa RP. Predictive Low-Glucose Suspend Reduces Hypoglycemia in Adults, Adolescents, and Children With Type 1 Diabetes in an At-Home Randomized Crossover Study: Results of the PROLOG Trial. Diabetes Care (2018) 41:2155–61. doi: 10.2337/dc18-0771 30089663

[22] Chen E King F Kohn MA Spanakis EK Breton M Klonoff DC. A Review of Predictive Low Glucose Suspend and Its Effectiveness in Preventing Nocturnal Hypoglycemia. Diabetes Technol Ther (2019) 21:602–09. doi: 10.1089/dia.2019.0119 31335193

[23] Hovorka R Chassin LJ Wilinska ME Canonico V Akwi JA Federici MO. Closing the Loop: The Adicol Experience. Diabetes Technol Ther (2004) 6:307–18. doi: 10.1089/152091504774197990 15198833

[24] Weinzimer SA Steil GM Swan KL Dziura J Kurtz N Tamborlane WV. Fully Automated Closed-Loop Insulin Delivery Versus Semiautomated Hybrid Control in Pediatric Patients With Type 1 Diabetes Using an Artificial Pancreas. Diabetes Care (2008) 31:934–39. doi: 10.2337/dc07-1967 18252903

[25] Kovatchev BP Renard E Cobelli C Zisser HC Keith-Hynes P Anderson SM. Safety of Outpatient Closed-Loop Control: First Randomized Crossover Trials of a Wearable Artificial Pancreas. Diabetes Care (2014) 37:1789–96. doi: 10.2337/dc13-2076 PMC4067397 24929429

[26] Thabit H Tauschmann M Allen JM Leelarathna L Hartnell S Wilinska ME. Home Use of an Artificial Beta Cell in Type 1 Diabetes. N Engl J Med (2015) 373:2129–40. doi: 10.1056/NEJMoa1509351 PMC4697362 26379095

[27] Bergenstal RM Garg S Weinzimer SA Buckinham BA Bode BW Tamborlane WV. Safety of a Hybrid Closed-Loop Insulin Delivery System in Patients With Type 1 Diabetes. JAMA (2016) 316:1407–08. doi: 10.1001/jama.2016.11708 27629148

[28] Garg SK Weinzimer SA Tamborlane WV Buckingham BA Bode BW Bailey TS. Glucose Outcomes With the In-Home Use of a Hybrid Closed-Loop Insulin Delivery System in Adolescents and Adults With Type 1 Diabetes. Diabetes Technol Ther (2017) 19:155–63. doi: 10.1089/dia.2016.0421 PMC5359676 28134564

[29] Hartnell S Fuchs J Boughton CK Hovorka R. Closed-Loop Technology: A Practical Guide. Pract Diabetes (2021) 38:33–9. doi: 10.1002/pdi.2350

[30] Boughton CK Hovorka R. Automated Insulin Delivery in Adults. Endocrinol Metab Clin North Am (2020) 49:167–78. doi: 10.1016/j.ecl.2019.10.007 PMC6986367 31980116

[31] Forlenza GP Pinhas-Hamiel O Liljenquist DR Shulman DI Bailey TS Bode BW. Safety Evaluation of the MiniMed 670g System in Children 7-13 Years of Age With Type 1 Diabetes. Diabetes Technol Ther (2019) 21:11–9. doi: 10.1089/dia.2018.0264 PMC6350071 30585770

[32] Lal RA Basina M Maahs DM Hood K Buckingham B Wilson DM. One Year Clinical Experience of the First Commercial Hybrid Closed-Loop System. Diabetes Care (2019) 42:2190–96. doi: 10.2337/dc19-0855 PMC6868462 31548247

[33] Da Silva J Lepore G Battelino T Arrieta A Castañeda J Grossman B. Real-World Performance of the MiniMed™ 670G System in Europe. Diabetes Obes Metab (2021) 23:1942–49. doi: 10.1111/dom.14424 33961340

[34] Lewis D. History and Perspective on DIY Closed Looping. J Diabetes Sci Technol (2019) 13:790–93. doi: 10.1177/1932296818808307 PMC6610599 30348013

[35] Jennings P Hussain S. Do-It-Yourself Artificial Pancreas Systems: A Review of the Emerging Evidence and Insights for Healthcare Professionals. J Diabetes Sci Technol (2020) 14:868–77. doi: 10.1177/1932296819894296 PMC7753866 31847570

[36] Palmer W Greeley SAW Naylor R. The Do-It-Yourself Artificial Pancreas. Pediatr Ann (2021) 50:e304–07. doi: 10.3928/19382359-20210622-02 34264792

[37] Asarani NAM Reynolds AN Elbalshy M Burnside M de Bock M Lewis DM. Efficacy, Safety, and User Experience of DIY or Open-Source Artificial Pancreas Systems: A Systematic Review. Acta Diabetol (2021) 58:539–47. doi: 10.1007/s00592-020-01623-4 33128136

[38] Toffanin C Kozak M Sumnik Z Cobelli C Petruzelkova L. In Silico Trials of an Open-Source Android-Based Artificial Pancreas: A New Paradigm to Test Safety and Efficacy of Do-It-Yourself Systems. Diabetes Technol Ther (2020) 22:112–20. doi: 10.1089/dia.2019.0375 31769699

[39] Armiger R Reddy M Oliver NS Georgiou P Herrero P. An In Silico Head-To-Head Comparison of the Do-It-Yourself Artificial Pancreas Loop and Bio-Inspired Artificial Pancreas Control Algorithms. J Diabetes Sci Technol (2022) 16:29–39. doi: 10.1177/19322968211060074 34861785 PMC8875066

[40] Messori M Cobelli C Magni L. Artificial Pancreas: From in-Silico to in-Vivo. IFAC-PapersOnLine (2015) 48:1300–08. doi: 10.1016/j.ifacol.2015.09.148

[41] Kesavadev J Srinivasan S Saboo B Krishna BM Krishnan G. The Do-It-Yourself Artificial Pancreas: A Comprehensive Review. Diabetes Ther (2020) 11:1217–35. doi: 10.1007/s13300-020-00823-z PMC7261300 32356245

[42] Phillip M Battelino T Atlas E Kordonouri O Bratina N Miller S. Nocturnal Glucose Control With an Artificial Pancreas at a Diabetes Camp. N Engl J Med (2013) 368:824–33. doi: 10.1056/NEJMoa1206881 23445093

[43] Russell SJ El-Khatib FH Sinha M Magyar KL McKeon K Goergen KL. Outpatient Glycemic Control With a Bionic Pancreas in Type 1 Diabetes. N Engl J Med (2014) 371:313–25. doi: 10.1056/NEJMoa1314474 PMC4183762 24931572

[44] Stewart ZA Wilinska ME Hartnell S Temple RC Rayman G Stanley KP. Closed-Loop Insulin Delivery During Pregnancy in Women With Type 1 Diabetes. N Engl J Med (2016) 375:644–54. doi: 10.1056/NEJMoa1602494 27532830

[45] Kovatchev B Cheng P Anderson SM Pinsker JE Boscari F Buckingham BA. Feasibility of Long-Term Closed-Loop Control: A Multicenter 6-Month Trial of 24/7 Automated Insulin Delivery. Diabetes Technol Ther (2017) 19:18–24. doi: 10.1089/dia.2016.0333 27982707

[46] El-Khatib FH Balliro C Hillard MA Magyar KL Ekhlaspour L Sinha M. Home Use of a Bihormonal Bionic Pancreas Versus Insulin Pump Therapy in Adults With Type 1 Diabetes: A Multicentre Randomised Crossover Trial. Lancet (2017) 389:369–80. doi: 10.1016/S0140-6736(16)32567-3 PMC5358809 28007348

[47] Brown SA Kovatchev BP Raghinaru D Lum JW Buckingham BA Kudva YC. Six-Month Randomized, Multicenter Trial of Closed-Loop Control in Type 1 Diabetes. N Engl J Med (2019) 381:1707–17. doi: 10.1056/NEJMoa1907863 PMC7076915 31618560

[48] Tauschmann M Allen JM Nagl K Fritsch M Yong J Metcalfe E. Home Use of Day-And-Night Hybrid Closed-Loop Insulin Delivery in Very Young Children: A Multicenter, 3-Week, Randomized Trial. Diabetes Care (2019) 42:594–600. doi: 10.2337/dc18-1881 30692242

[49] Boughton CK Hovorka R. New Closed-Loop Insulin Systems. Diabetologia (2021) 64:1007–15. doi: 10.1007/s00125-021-05391-w PMC8012332 33550442

[50] Look H. 99 Years Closer: About Our FDA Submission of Tidepool Loop (2021). Tidepool. Available at: https://www.tidepool.org/blog/99-years-closer-about-our-fda-submission-of-tidepool-loop (Accessed April 14, 2022).

[51] Weisman A Bai JW Cardinez M Kramer CK Perkins BA. Effect of Artificial Pancreas Systems on Glycaemic Control in Patients With Type 1 Diabetes: A Systematic Review and Meta-Analysis of Outpatient Randomised Controlled Trials. Lancet Diabetes Endocrinol (2017) 5:501–12. doi: 10.1016/S2213-8587(17)30167-5 28533136

[52] Samadi S Rashid M Turksoy K Feng J Hajizadeh I Hobbs N. Automatic Detection and Estimation of Unannounced Meals for Multivariable Artificial Pancreas System. Diabetes Technol Ther (2018) 20:235–46. doi: 10.1089/dia.2017.0364 PMC5867514 29406789

[53] Agianniotis A Anthimopoulos M Daskalaki E Drapela A Stettler C Diem P. GoCARB in the Context of an Artificial Pancreas. J Diabetes Sci Technol (2015) 9:549–55. doi: 10.1177/1932296815583333 PMC4604547 25904142

[54] Kovatchev B Cobelli C Renard E Anderson S Breton M Patek S. Multinational Study of Subcutaneous Model-Predictive Closed-Loop Control in Type 1 Diabetes Mellitus: Summary of the Results. J Diabetes Sci Technol (2010) 4:1374–81. doi: 10.1177/193229681000400611 PMC3005047 21129332

[55] Forlenza GP Cameron FM Ly TT Lam D Howsmon DP Baysal N. Fully Closed-Loop Multiple Model Probabilistic Predictive Controller Artificial Pancreas Performance in Adolescents and Adults in a Supervised Hotel Setting. Diabetes Technol Ther (2018) 20:335–43. doi: 10.1089/dia.2017.0424 PMC5963546 29658779

[56] Cameron FM Ly TT Buckingham BA Maahs DM Forlenza GP Levy CL. Closed-Loop Control Without Meal Announcement in Type 1 Diabetes. Diabetes Technol Ther (2017) 19:527–32. doi: 10.1089/dia.2017.0078 PMC5647490 28767276

[57] Cherñavvsky DR DeBoer MD Keith-Hynes P Mize B McElwee M Demartini S. Use of an Artificial Pancreas Among Adolescents for a Missed Snack Bolus and an Underestimated Meal Bolus. Pediatr Diabetes (2016) 17:28–35. doi: 10.1111/pedi.12230 25348683

[58] Tagougui S Taleb N Molvau J Nguyen É Raffray M Rabasa-Lhoret R. Artificial Pancreas Systems and Physical Activity in Patients With Type 1 Diabetes: Challenges, Adopted Approaches, and Future Perspectives. J Diabetes Sci Technol (2019) 13:1077–90. doi: 10.1177/1932296819869310 PMC6835182 31409125

[59] DeBoer MD Cherñavvsky DR Topchyan K Kovatchev BP Francis GL Breton MD. Heart Rate Informed Artificial Pancreas System Enhances Glycemic Control During Exercise in Adolescents With T1D. Pediatr Diabetes (2017) 18:540–46. doi: 10.1111/pedi.12454 27734563

[60] Namikawa T Munekage M Yatabe T Kitagawa H Hanazaki K. Current Status and Issues of the Artificial Pancreas: Abridged English Translation of a Special Issue in Japanese. J Artif Organs (2018) 21:132–37. doi: 10.1007/s10047-018-1019-4 29356912

[61] Bakhtiani PA Zhao LM El Youssef J Castle JR Ward WK. A Review of Artificial Pancreas Technologies With an Emphasis on Bi-Hormonal Therapy. Diabetes Obes Metab (2013) 15:1065–70. doi: 10.1111/dom.12107 PMC3766424 23602044

[62] Peters TM Haidar A. Dual-Hormone Artificial Pancreas: Benefits and Limitations Compared With Single-Hormone Systems. Diabetes Med (2018) 35:450–59. doi: 10.1111/dme.13581 29337384

[63] Blauw H van Bon AC Koops R DeVries JH. On Behalf of the PCDIAB Consortium. Performance and Safety of an Integrated Bihormonal Artificial Pancreas for Fully Automated Glucose Control at Home. Diabetes Obes Metab (2016) 18:671–77. doi: 10.1111/dom.12663 PMC5111773 26996542

[64] Trevitt S Simpson S Wood A. Artificial Pancreas Device Systems for the Closed-Loop Control of Type 1 Diabetes: What Systems Are in Development? J Diabetes Sci Technol (2016) 10:714–23. doi: 10.1177/1932296815617968 PMC5038530 26589628

[65] Castellanos LE Balliro CA Sherwood JS Jafri R Hillard MA Greaux E. Performance of the Insulin-Only Ilet Bionic Pancreas and the Bihormonal Ilet Using Dasiglucagon in Adults With Type 1 Diabetes in a Home-Use Setting. Diabetes Care (2021) 44:e118–20. doi: 10.2337/dc20-1086 PMC8247518 33906916

[66] Sherr JL Patel NS Michaud CI Palau-Collazo MM Van Name MA Tamborlane WV. Mitigating Meal-Related Glycemic Excursions in an Insulin-Sparing Manner During Closed-Loop Insulin Delivery: The Beneficial Effects of Adjunctive Pramlintide and Liraglutide. Diabetes Care (2016) 39:1127–34. doi: 10.2337/dc16-0089 PMC4915555 27208332

[67] Haidar A Tsoukas MA Bernier-Twardy S Yale J Rutkowski J Bossy A. A Novel Dual-Hormone Insulin-And-Pramlintide Artificial Pancreas for Type 1 Diabetes: A Randomized Controlled Crossover Trial. Diabetes Care (2020) 43:597–606. doi: 10.2337/dc19-1922 31974099

[68] Riddle MC. Rediscovery of the Second β-Cell Hormone: Co-Replacement With Pramlintide and Insulin in Type 1 Diabetes. Diabetes Care (2020) 43:518–21. doi: 10.2337/dci19-0077 32079687

[69] Fuchs J Hovorka R. Benefits and Challenges of Current Closed-Loop Technologies in Children and Young People With Type 1 Diabetes. Front Pediatr (2021) 9:679484. doi: 10.3389/fped.2021.679484 33996702 PMC8119627

[70] Polsky S Akturk HK. Case Series of a Hybrid Closed-Loop System Used in Pregnancies in Clinical Practice. Diabetes Metab Res Rev (2020) 36:e3248. doi: 10.1002/dmrr.3248 31758630

[71] Guzmán Gómez GE Viggiano JA Silva-De Las Salas A Martínez V Urbano Bonilla MA. The Closed-Loop System Improved the Control of a Pregnant Patient With Type 1 Diabetes Mellitus. Case Rep Endocrinol (2021) 2021, 7310176. doi: 10.1155/2021/7310176 34594581 PMC8478568

[72] Stewart ZA Wilinska ME Hartnell S O'Neil LK Rayman G Scott EM. Day-And-Night Closed-Loop Insulin Delivery in a Broad Population of Pregnant Women With Type 1 Diabetes: A Randomized Controlled Crossover Trial. Diabetes Care (2018) 41:1391–99. doi: 10.2337/dc17-2534 29535135

[73] Bally L Thabit H Hartnell S Andereggen E Ruan Y Wilinska ME. Closed-Loop Insulin Delivery for Glycemic Control in Noncritical Care. N Engl J Med (2018) 379:547–56. doi: 10.1056/NEJMoa1805233 29940126

[74] Bally L Gubler P Thabit H Hartnell S Ruan Y Wilinska ME. Fully Closed-Loop Insulin Delivery Improves Glucose Control of Inpatients With Type 2 Diabetes Receiving Hemodialysis. Kidney Int (2019) 96:593–96. doi: 10.1016/j.kint.2019.03.006 31133457

[75] Ware J Allen JM Boughton CK Wilinska ME Hartnell S Thankamony A. Randomized Trial of Closed-Loop Control in Very Young Children With Type 1 Diabetes. N Engl J Med (2022) 386:209–19. doi: 10.1056/NEJMoa2111673 35045227

